# Role of Dietary Supplements in the Management of Parkinson’s Disease

**DOI:** 10.3390/biom9070271

**Published:** 2019-07-10

**Authors:** Michele Ciulla, Lisa Marinelli, Ivana Cacciatore, Antonio Di Stefano

**Affiliations:** Department of Pharmacy, University “G. d’Annunzio” of Chieti-Pescara, via dei Vestini 31, 66100 Chieti Scalo (CH), Italy

**Keywords:** Parkinson’s disease, food supplements, functional food, antioxidants, anti-inflammatory, neuroprotection, natural compounds

## Abstract

The use of food supplements or functional food has significantly increased in the past decades, especially to compensate both the modern lifestyle and the food shortages of the industrialized countries. Despite food supplements are habitually intended to correct nutritional deficiencies or to support specific physiological functions, they are often combined with common drug therapies to improve the patient’s health and/or mitigate the symptoms of many chronic diseases such as cardiovascular diseases, cystic fibrosis, cancer, liver and gastrointestinal diseases. In recent years, increased attentions are given to the patient’s diet, and the use of food supplements and functional food rich in vitamins and antioxidants plays a very important role in the treatment and prevention of neurodegenerative diseases such as Parkinson’s disease (PD). Natural compounds, phytochemicals, vitamins, and minerals can prevent, delay, or alleviate the clinical symptoms of PD in contrast to some of the main physiopathological mechanisms involved in the development of the disease, like oxidative stress, free radical formation, and neuroinflammation. The purpose of this review is to collect scientific evidences which support the use of specific biomolecules and biogenic elements commonly found in food supplements or functional food to improve the clinical framework of patients with PD.

## 1. Introduction

The use of food supplements has significantly increased over the past decades, especially in the industrialized countries, with a trend that is expected to increase for the coming years [1]. The reasons for this growth can be ascribed primarily to the modern times, the industrialization process, and the food sources, which are now poorer in important nutrients. Moreover, the frenetic lifestyle that people tend to adopt, as well as the increase of life expectancy and the incidence in chronic diseases provide a constant attention to the intake of specific nutritional elements to compensate for food shortages.

In recent years, a positive outlook toward the medical nutrition market was assessed, estimating that the constant use of food supplements has led to the global dietary supplements market at $133.1 billion in 2016 and is projected to accelerate at CAGR (compound annual growth rate) of 9.6%, reaching $278.02 billion by 2024 [1]. Vitamins-based supplements are projected to account for 48% of the global share by the end of 2024. The European Food Safety Authority (EFSA) defines food supplements as a “*concentrated source of nutrients or other substances with a nutritional or physiological effect that are marketed in dose form. A wide range of nutrients and other ingredients might be present in food supplements, including, but not limited to, vitamins, minerals, amino acids, essential fatty acids, fiber and various plants and herbal extracts*” [2]. In this context, the role of food supplements is to compensate nutritional deficits through an appropriate consumption of specific components, thus supporting several biological processes. It is important to underline that these products are not medicines, since they cannot simulate any pharmacological activity and treat or prevent the onset of diseases [3]. Indeed, claims relating to food supplements are strictly regulated by EFSA, which only after a deep evaluation of scientific literature allows to indicate in which terms the vitamin or more in general the nutrient in question can exert beneficial nutritional effects [4].

Nevertheless, nutritional supplements are often combined with pharmacological therapies for many chronic diseases such as cardiovascular diseases, cystic fibrosis, cancer, human immunodeficiency virus and acquired immune deficiency syndrome, liver and gastrointestinal diseases, and nutritional status related diseases [5,6]. Moreover, nutrition also plays a very important role in the treatment and prevention of neurodegenerative diseases, especially in the older age groups [7]. Recently, it has been highlighted that the consumption of functional food and food supplements can contribute greatly in the management of age-related diseases such as Parkinson’s disease (PD) [8]. Indeed, natural compounds, phytochemicals, vitamins, minerals present in the food supplements or fruits, vegetables, and spices can prevent, delay, or alleviate the clinical symptoms of chronic neurodegenerative diseases, improving cognitive functions, learning, general brain status, and wellbeing [9].

The purpose of this review is to collect scientific evidences to support the use of specific biomolecules commonly found in food supplements or functional food in order to improve the clinical framework of patients with PD. By analyzing scientific literature data, it will be possible to identify which vitamins, minerals, or different molecules have shown to exert potential properties useful to counteract or mitigate physiopathological phenomena related to PD, such as oxidative stress and neuroinflammation.

## 2. Parkinson’s Disease

**Figure 1:**
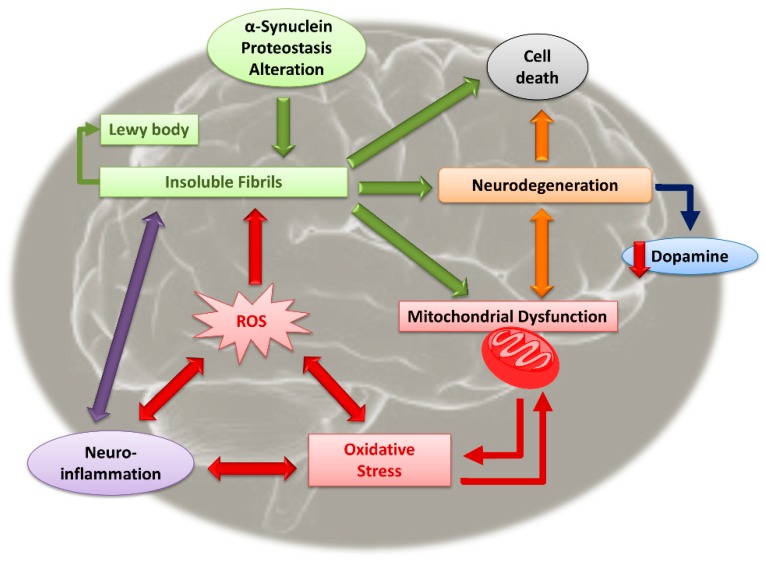
Interaction between the major molecular mechanism involved in the pathogenesis of Parkinson’s disease (PD).

PD is a degenerative neurological disorder characterized by the onset motor symptoms such as tremors, muscle rigidity, slowness in movement (bradykinesia), and stooped posture (postural instability) [10]. Moreover, non-motor symptoms are also present, including disorders of mood and affect, apathy, anhedonia and depression, cognitive dysfunction and hallucinosis, sleep disruption, as well as complex behavioral disorders. PD affects 1–2‰ of the population at any time, and together with Alzheimer’s disease (AD), is one of the most common neurologic disorders [11]. PD affects approximately 1% of individuals older than 60 years, with an increment in the incidence of 5–10 times from the sixth to the ninth decade of life [12]. Environmental and genetic factors are both involved in the onset of PD, even if the framework of the disease is not completely elucidated [13]. Regarding the neuropathological mechanisms, the major evidence is the loss of dopaminergic neurons of the substantia nigra pars compacta and the locus coeruleus. The first is responsible for motor control, while the latter is responsible for various psychological effects. One of the most important consequences of neuronal degeneration is the reduction of dopamine (DA), responsible for numerous biological functions [14]. Furthermore, a brain affected by PD is characterized by the presence of Lewy bodies and Lewy neurites [15]. Lewy bodies consist of protein agglomerations (α-synuclein, parkin, and other proteins) embedded in the cytoplasm of dead neurons located in several different brain regions. Agglomeration of α-synuclein starts when soluble monomers initially form oligomers, then progressively continue to merge and thus form large, insoluble fibrils [16]. Beyond these two major clinical evidences, PD involves different molecular mechanisms, all related to each other and directly linked to the physiopathology of the disease, in a sort of vicious circle that exacerbate the neurodegeneration process (Figure 1).

There are evidences that correlate neuronal mitochondrial dysfunction with the pathogenesis of PD [17,18]. This dysfunction is associated with the abnormal accumulation of α-synuclein, which causes an alteration of normal mitochondrial function, leading to neuronal degeneration and strong oxidative stress [19,20]. The latter is exacerbated in the nervous tissue of patients with PD, especially at the level of nigral dopaminergic neurons, particularly vulnerable to oxidative and metabolic stress [21,22]. The presence of neuroinflammation is another peculiar characteristic of PD, which plays an important role in the progression of the disease. The inflammation depends also on the impaired energy metabolism at the level of the mitochondria, which causes the activation of the microglia and the relative production of a plethora of pro-inflammatory mediators, including prostaglandins, cytokines, chemokines, complement, proteinases, reactive species of oxygen (ROS), and reactive species of nitrogen (RNS) [23,24].

All these evidences lead to the degeneration of dopaminergic neurons, a reduced dopaminergic transmission in the motor region of the striatum, and therefore cell death [22]. Moreover, PD causes the alteration of two other neurotransmission complexes such as the cholinergic and the serotonergic systems. In particular, a reduction in the cholinergic and muscarinic receptors was highlighted in patients with PD, with negative consequences related to cognitive and motor functions [25]. The diagnosis of PD is difficult to define before the symptoms of the disease become evident [26]. Moreover, most patients with PD also have non-motor symptoms, including disorders of sleep–wake cycle regulation, cognitive impairment disorders of mood and affect, autonomic dysfunction, as well as sensory and pain [27]. The treatments available today aim to restore the level of dopamine in the striatum in order to contain the motor disorders, and levodopa (L-DOPA) is still the gold standard for the treatment of parkinsonism [28]. Treatment with L-DOPA, accompanied by a series of pharmacological strategies, aims to prevent peripheral dopamine metabolism and improve its bioavailability, thus reducing motor complications due to its administration, as well as further therapies to control non-motor symptoms mentioned before that weigh on the quality of life of the patients with PD [29,30,31]. In recent years, medical therapies have been flanked by non-pharmacological alternatives such as exercises, group therapies, nutrition and food supplementation [32,33,34]. Recently, the management of age-related diseases such as PD has been associated with consumption of functional food or food supplements. Indeed, a healthy diet rich in foods containing antioxidants, vitamins and minerals, or the use of food supplements can help to reduce and/or contrast the symptoms of PD and the related pathological mechanisms [35,36].

## 3. Oxidative Stress and Neuroinflammation in PD

Oxidative stress, generation of free radicals, and generation of ROS in neurons and glial cells contribute and play a major role in the pathogenesis of PD and other neurodegenerative diseases [21,37]. In particular, nigral dopaminergic neurons seem to be particularly vulnerable to oxidative stress mainly because of the long axons and millions of synapses, which require high energy to work properly [38]. Besides high energy, increased levels of dopamine and its metabolites can also cause oxidative stress [39]. Finally, the mitochondrial dysfunction naturally leads to an increased level of oxidative stress, with a depletion of lysosome and the establishment of a vicious cycle in which the first increases the alteration of the latter and vice versa [40].

At the same time, the antioxidant defensive mechanism in the brain is poor and thus limits its protective action against free radical attack, especially during the progression of neurodegenerative conditions, in which the cell’s antioxidant capacity is further reduced. Antioxidants are able to scavenge free radicals and reactive species, giving a beneficial therapeutic effect in PD by preventing the onset of apoptotic cell death and neuronal degeneration of the dopaminergic nigrostriatal pathway.

On the same level, neuroinflammation plays an important role in the pathogenesis of PD, highlighting an increased activation of microglia and the amplification of proinflammatory and toxic mediators [41]. In particular, the presence of α-synuclein insoluble fibrils activates glial cells and other immune/inflammatory cells, with the subsequent release of inflammatory cytokines, chemokines, and neurotoxic mediators, which induce neurodegeneration in dopaminergic neurons [42]. Moreover, toxic insoluble fibrils also have direct proinflammatory activity in PD, contributing to neurodegeneration and neuronal cell death (Figure 1) [43]. Finally, peripheral immune and inflammatory cells migrate to the brain through the defective blood brain barrier (BBB), increasing neuroinflammation either directly or through glial and neuronal cells [41]. The intake of anti-inflammatory compounds from food supplements or functional food can lead to the suppression or reduction of neuroinflammation, ameliorating PD symptoms and reducing the extent of neurodegeneration.

## 4. Natural Compounds Useful in the Prevention and Management of PD

Scientific evidences have shown that numerous molecules and natural compounds are able to mitigate the symptoms of PD by counteracting the physiopathological mechanisms which dominate the disease, such as oxidative stress and neuroinflammation. Furthermore, some molecules have shown to possess neuroprotective and neuro-modulatory properties.

Table 1 displays the analyzed molecules, describing for each compound the beneficial effects demonstrated experimentally and the performed mechanism that support a positive incidence in the treatment of PD.

### 4.1. Coenzyme Q10

**Figure 2:**
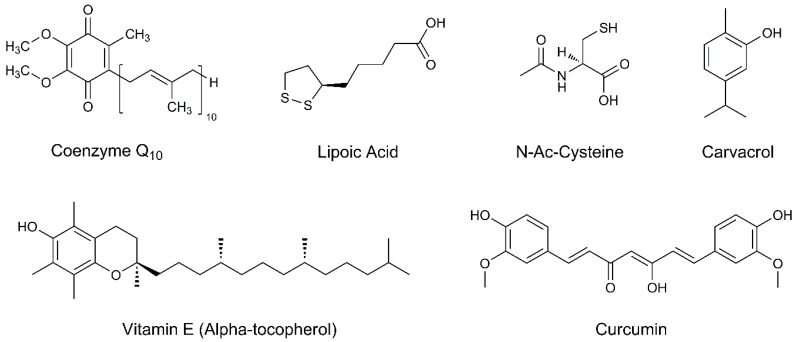
Chemical structures of the examined natural compounds.

Coenzyme Q10 (CoQ10, Figure 2), also known as ubiquinone, is a 1,4-benzoquinone that is ubiquitous in animals and most bacteria. Its natural sources are foods such as tuna or salmon, organ meats, and whole grains, but recently food supplements rich in CoQ10 have also become popular. CoQ10 is a component of the electron transport chain and participates in the aerobic cellular respiration, which generates energy in the form of ATP. This molecule acts not only as an important electron carrier in the electron transport chain, but also as a free-radical scavenging antioxidant [44]. In particular, several in vitro studies showed that CoQ10 has neuroprotective effects in multiple models of neuronal toxicity. It was also highlighted that oral supplementation of CoQ10 can reduce the loss of dopamine and dopaminergic axons in the striatum in 1-year-old mice treated with MPTP-induced mouse model of PD (1-methyl-4-phenyl-1,2,3,6-tetrahydropyridine, MPTP) [45,46]. Nevertheless, the role of CoQ10 in the management of PD is controversial.

Shults and collaborators in 2002 conducted a phase II trial, comparing placebo and three dosages of CoQ10 (300, 600, and 1200 mg/day) in a prospective, randomized, double-blind study with 20 subjects in each group [47]. The authors found that the high dosages of CoQ10 were safe and well tolerated, with a reduction of disability for patients treated with the coenzyme compared to placebo, inducing a slowdown of the progressive deterioration of function in PD.

Moreover, Muller et al. in 2003 performed a monocenter, parallel group, placebo-controlled, double-blind trial to determine the symptomatic response of daily oral application of 360 mg CoQ10 during 4 weeks of treatments [48]. Results suggested that an oral CoQ10 supplementation provides moderate beneficial effect in PD patients.

On the contrary, Storch et al. in 2007 performed a clinical study applying the same protocol and the same maximum dosage as of the Shults’ study (1200 mg/day) but did not find any appreciable benefits in mid-stage PD after three months of treatment [49].

More recently, meta-analysis was done to highlight the qualitative and quantitative conclusions about the efficacy of CoQ10 for the treatment of PD [50]. Final results showed that CoQ10 was safe and well tolerated in PD patients but did not present evidence of clinical benefits. Additional trials are required to confirm the role of CoQ10 in slowing the progressive deterioration of function in PD.

### 4.2. Lipoic Acid

Lipoic acid (LA, Figure 2) is a compound naturally synthesized in the human body and also found in several foods. It has potential therapeutic value since it exhibits antioxidative and anti-inflammatory activity, as well as it inhibits free radical formation [51].

Zhang et al. in 2018 investigated not only the antioxidant and anti-inflammatory properties of LA in an in vivo mouse model of PD, but they also assessed the capacity of LA to reduce the dyskinesia side effects related to the L-DOPA administration [52]. The 6-OHDA-lesioned rats (6-hydroxydopamine, 6-OHDA) were treated with LA (31.5 mg/kg or 63 mg/kg) in combination with L-DOPA treatment, highlighting that LA reduces L-DOPA-induced dyskinesia in a dose-dependent manner without compromising the anti-PD effect of the drug. Moreover, LA reduced the level of malondialdehyde, a product of lipid peroxidation, and upregulated the glutatione (GSH) activity, a clear indication of the positive antioxidant effects. The authors concluded that LA can be recommended as a promising disease-modifying therapy when administered with L-DOPA.

Jalali-Nadoushan and Roghani investigated the effect of this compound (at doses of 50 and 100 mg/kg) in a 6-OHDA-induced mouse model of PD, highlighting that LA significantly attenuated rotations on behavioral testing in particular at a dose of 100 mg [53]. These results confirm that LA can partially afford neuroprotection against 6-OHDA neurotoxicity due to the attenuation of oxidative stress burden.

Li et al. tested the antioxidant and anti-inflammatory properties of LA on a lipopolysaccharide (LPS)-induced inflammatory PD model [54]. After the treatment with LPS, in order to induce the microglia activation, mice were treated with LA once a day at 100 mg/kg. Results showed important improvement in motor dysfunctions, a reduction in α-synuclein accumulation, and a reduction of the pro-inflammatory molecules activation, suggesting that LA may exert a significant neuroprotective, anti-neuroinflammatory, and anti-oxidative effect, thus becoming a promising agent for halting the progression of PD.

### 4.3. N-Acetyl-Cysteine

*N*-acetyl-cysteine (NAC, Figure 2) is an N-acetylated derivative of the sulphurated cysteine amino acid. The -SH group is able to actively contrast ROS, conferring antioxidant properties to the molecule [55]. At the same time, NAC and its analogues contribute to the physiological antioxidant activity by acting as a GSH precursor [56,57].

Potential protective properties of NAC in the management of PD were assessed in animal model studies, demonstrating a sensible reduction of oxidative damage by increasing mitochondrial complex I and IV activities and preventing ROS accumulation, leading in this way to the protection of dopamine-induced cell death [58].

Holmay et al. in 2013 investigated the potential antioxidant properties of NAC by measuring the level of GSH in patients with PD before and after its administration [59]. Results showed that after intravenous injection of NAC, there was a boost in antioxidant GSH levels in the brain and blood of PD patients, making possible the compensation of the hypothesized deficiency and lower GSH activity in PD.

Monti et al. in 2016 highlighted the potential protective properties of NAC in PD using an in vitro and in vivo model [60]. The first model revealed an increased dopaminergic neurons survival in cells treated with rotenone compared to placebo. The clinical study confirmed the protective effects previously observed with an increased dopamine transporter binding in the caudate and putamen in the PD group treated with NAC, and no measurable changes in the control group.

### 4.4. Vitamin E

Vitamin E is a group of eight fat soluble compounds that include four tocopherols and four tocotrienols; abundant in vegetable oils, whole-grain cereals, butter, and eggs. They are involved in several human biological functions, with the alpha-tocopherol (Figure 2) as the main form of vitamin E, preferentially absorbed and accumulated in human body. It acts as an antioxidant, scavenger of several ROS, including hydroxyl and peroxyl radicals, and it is able to inhibit lipid peroxidation [61]. Some clinical trials were carried out to better understand the potential neuroprotective properties of this molecule in the management of PD, but the results are controversial [62].

Fahn tried to administer a combination of ascorbate and vitamin E to patients with early PD in a first pilot open-labeled trial [63]. The primary end point of the trial was the progression of the disease until patients needed treatment with L-DOPA or a dopamine agonist compared to control, and results showed that patients who received the antioxidants combination extended the time when L-DOPA became necessary by 2.5 years, suggesting that the progression of PD may be slowed by the administration of vitamin C and E.

Zhang et al. documented the occurrence of PD within two large cohorts of men and women who completed detailed and validated semiquantitative food frequency questionnaires, highlighting that supplementation with antioxidant vitamins is not associated with the risk of PD, but it is significantly reduced among men and women with high intake of dietary vitamin E [64].

Nevertheless, in another clinical trial, Scheider and collaborators took into consideration the possible role of long-term dietary antioxidants intake in PD onset [65]. Results showed that vitamin E did not show evidence of benefits in either improving the clinical features of PD or delaying the functional decline.

A large clinical trial was conducted to examine the benefits of deprenyl (selegiline) and alpha-tocopherol in slowing the progression of PD (DATATOP study, Deprenyl and tocopherol antioxidative therapy of parkinsonism) [66]. The observation indicated that deprenyl delay the time of disability development, while alpha-tocopherol produced no benefits. Additional trials are still needed to confirm the role of vitamin E in slowing the progressive deterioration of function in PD. 

### 4.5. Carvacrol

Carvacrol (Figure 2) is a phenolic monoterpene found in numerous aromatic plants including basil, rosemary, thyme, and oregano. The last possesses the highest percentage of carvacrol-enriched essential oil. Several studies investigated the properties of this monoterpene, highlighting the numerous pharmacological properties, including antibacterial, antifungal, anti-inflammatory, antioxidant, and neuro-modulatory action [67,68]. As a neuro-modulatory agent, the ability to also regulate the activity of dopaminergic neurons has made the use of carvacrol for the treatment of PD interesting.

Haddadi et al. experimentally assessed the effect of carvacrol in a mouse model of PD [69]. The animals were treated with 6-OHDA to induce the onset of the typical symptoms of PD, and subsequently different tests were carried out to determine the motor and cognitive abilities of the treated animals. In particular, the apomorphine-induced rotation test showed that treatment with carvacrol exerted no differences between the treated and the untreated group. Regarding the passive avoidance memory test, the treatment of mice with carvacrol at concentrations of 25 mg/kg showed clear improvements compared to the control group. Finally, the tail-flick test showed no difference between the carvacrol-treated group and the control. The authors concluded that carvacrol showed improved cognitive impairments without having effects on pain and motor symptoms. 

### 4.6. Curcumin

Curcumin (diferuloylmethane, Figure 2), is a polyphenol extracted from the rhizome of *Curcuma longa*, a plant widely distributed in Asia. The powder, derived from the rhizome, is used as a spice and as a natural remedy in traditional oriental medicine. The properties that curcumin boasts include antioxidant, anti-inflammatory, and neuroprotective activities, which can have a positive impact on the treatment of PD [70].

Wang et al. carried out an analysis of the scientific literature, collecting 113 studies concerning curcumin and PD [71]. Among these, specific inclusion criteria led to assessing 13 studies as relevant in relationship to the effects that curcumin can exert in animal models of PD. In particular, the three major findings that the authors described are that curcumin can perform anti-inflammatory, antioxidant, and antiapoptotic action. The anti-inflammatory properties were highlighted in five studies, where curcumin attenuated the DNA damage caused by numerous metals and reduced the presence of pro-inflammatory cytokines [72,73,74,75,76]. The antioxidant properties presented in several studies, demonstrated the ability of curcumin to reduce in vivo the ROS levels, lipid peroxidation, and NO generation [72,76,77]. Finally, the antiapoptotic properties have been identified in two studies, where curcumin reduced the pro-apoptotic cellular proteins level, conferring neuroprotection to the brain tissues in the examined animal models of PD [73,78].

### 4.7. Omega-3 Fatty Acids

**Figure 3:**
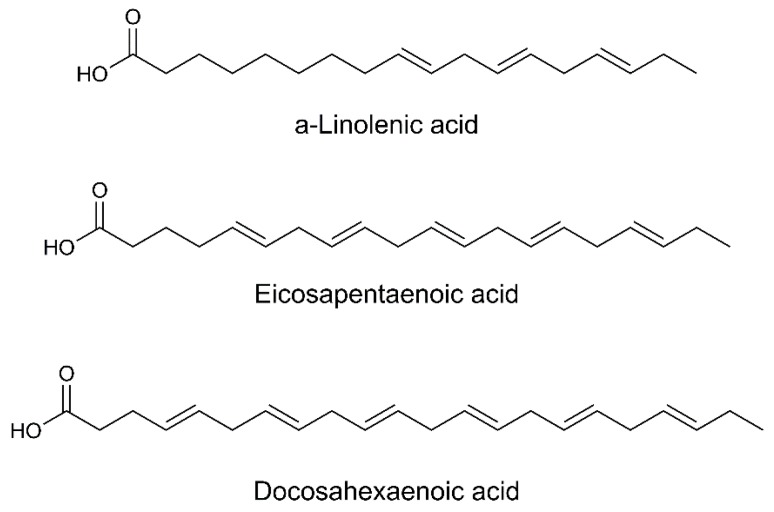
Chemical structures of the principal polyunsaturated fatty acids omega-3.

Omega-3 fatty acids have as main feature, a double bond, located three atoms far from the last methyl group, which is part of the backbone in their polyunsaturated structure (Figure 3). Omega-3 are present in certain foods such as cold-water fish (salmon) and fish oils, walnut, edible seeds and flaxseed oil, as well as dietary supplements in which fish oil is formulated as soft gel capsules. These types of fatty acids are important constituents of animal lipid metabolism, and they play an important role in the human diet and in human physiology, contributing to lower the levels of cholesterol and LDL (low-density lipoproteins) in the blood [79].

Beside these already known functions, Taghizadeh et al. evaluated the effects of omega-3 fatty acids and vitamin E co-supplementation on clinical signs and metabolic status in patient with PD, assessing a randomized clinical trial conduced in double-blind against a control group [80]. The treated group received omega-3 fatty acids (1000 mg) in combination with vitamin E (400 IU) for three months. Results showed that omega-3 fatty acids and vitamin E co-supplementation led to a significant improve in the selected rating scale used to assess the stage of PD. Furthermore, co-supplementation decreased high-sensitivity C-reactive protein and increased total antioxidant capacity compared with the placebo, demonstrating that omega-3 fatty acids and vitamin E co-supplementation in people with PD had favorable effects not only on the development of PD symptoms, but also in the management of ROS production and oxidative stress reduction. 

### 4.8. Whey Protein

Protein obtained by whey are a mixture of different lactoglobulins, serum albumin, and immunoglobulins, and more importantly, it is an excellent dietary source of cysteine. Several studies highlighted the capacity of whey protein to increase GSH, suggesting that this complex mixture is capable of boosting GSH synthesis, thus reducing oxidative stress [81].

Tosukhowong et al. in 2016 conducted a placebo-controlled, double-blind study on PD patients to investigate the effects of whey protein supplementation on plasma GSH, plasma amino acids, and the unified Parkinson’s disease rating scale modifications [82].

Results showed a significant increase in plasma concentration of reduced GSH, plasma branched chain amino acids (BCAA), and essential amino acids in the whey-supplemented group only. The rate of disease modification was not significantly ameliorated in either group, indicating that whey protein supplementation significantly increases plasma reduced glutathione in patients with PD with no significant changes in clinical outcomes.

### 4.9. Vitamin D_3_

**Figure 4:**
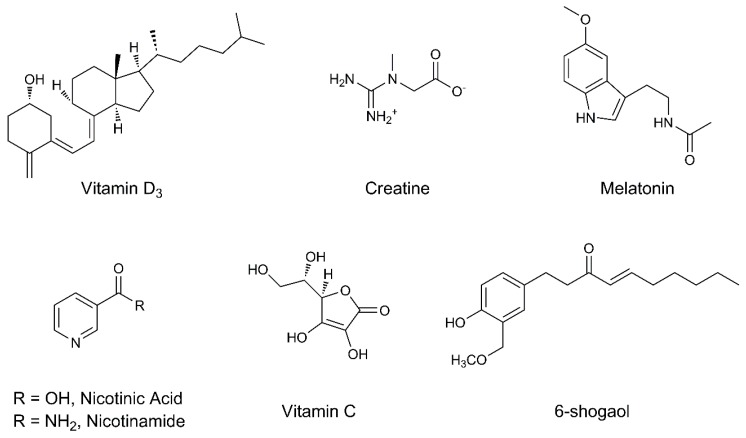
Chemical structures of the examined natural compounds.

Vitamin D_3_ (cholecalciferol, Figure 4) is endogenously produced when skin is exposed to the UV-B rays from the sun. Only a few foods such as cod liver oil, tuna, carp, salmon, fat cheeses, and mushrooms contain vitamin D_3_. Thus, it can be ingested directly from food supplements. Between the liposoluble steroids belonging to the group of vitamin D, the vitamin D_3_ is one of the most important since it is involved in different physiological processes, among them the most known is the calcium absorption and the bone growth regulation. Recently, several studies highlighted a possible implication in the muscular and endocrine apparatus, as well as in the central nervous system, with a positive attitude as neuroprotective agent in the management of PD [83].

Wang et al. studied the neuroprotective effects of vitamin D_3_ against 6-OHDA-lesioned mice in vivo and in vitro [84]. Results showed that the pretreatment with vitamin D_3_ for eight days significantly restored locomotor activity in the lesioned mice. Neurochemical analysis determined a protection from oxidative stress and reduction in depletion of DA neurons in mice treated with the vitamin compared to the control.

Jang et al. investigated the protective, autophagy-modulating effects of an active form of vitamin D₃ in an in vitro model of PD [85]. Result showed that the treatment with the vitamin reduced ROS levels and increased the levels of intracellular signaling proteins associated with cell survival.

Moreover, some clinical studies suggest that vitamin D_3_ has a positive effect on PD.

The first evidence was described by Evat et al. in a study of 2008, in which the level of vitamin D_3_, in 55% of patients with PD, was insufficient compared to 36% of a control population [86]. 

Knekt et al. carried out a cohort study on the Finnish population, collecting information regarding the onset of PD and measuring at the same time the levels of 25-hydroxy vitamin D, which is a serum indicator of the vitamin D_3_ concentration in the body, from 1978–1980 until the end of 2007 [87]. The results showed that higher levels of vitamin D_3_ are related to a lower risk of developing PD, giving an important indication of the potential neuroprotective activity of vitamin D_3_ against this disease.

### 4.10. Creatine

Creatine is a nitrogenous guanidine molecule that occurs naturally in vertebrates and helps to supply energy to muscle and nerve cells (Figure 4). This molecule usually is used by athletes to increase maximum power and performance in high-intensity anaerobic repetitive work, but there are also evidences of antioxidant properties, mitochondrial dysfunction reduction, and neuroprotective properties in in vitro and in vivo models of PD [88]. 

Matthews et al. assessed the neuroprotective effect of creatine in a MPTP-induced mouse model of PD [89]. Results showed that oral supplementation with either creatine or cyclocreatine produced significant protection against MPTP-induced dopamine depletions in mice, with a decreased dopaminergic neurons degeneration.

Yang et al. examined whether a combination of CoQ10 with creatine can exert additive neuroprotective effects in a MPTP mouse model of PD [90]. After administration of MPTP, the treatment with the two antioxidant molecules significantly reduced lipid peroxidation and pathologic α- synuclein accumulation in the neurons of the MPTP-treated mice, producing additive neuroprotective effects.

Despite these positive results, Bender et al. conducted a two-year placebo-controlled randomized clinical trial on the effect of creatine in 60 patients with PD, highlighting that creatine improves patient mood and led to a smaller dose increase of dopaminergic therapy but had no effect on the disease modification [91]. Further clinical trial may clarify clinical benefits of creatine in the treatment of PD.

### 4.11. Melatonin

Melatonin is a hormone produced in the pineal gland (Figure 4), and it is involved in synchronizing the circadian rhythms including sleep–wake timing, blood pressure regulation, and seasonal reproduction. Moreover, several studies described the capacity of melatonin to exert relevant antioxidant properties [92].

In particular, Antolin et al. described the capacity of melatonin to act as an antioxidant in a MPTP-induced mouse model of PD [93]. Results showed that melatonin can prevent neuronal cell death, contrasting the damage induced by chronic administration of MPTP, and preventing neuronal degeneration in the nigrostriatal pathway by counteracting induced oxidative and nitrative stress.

Dabbeni-Sala et al. demonstrated the neuroprotective action of melatonin in a 6-OHDA animal model of PD [94]. The authors observed the protective activity of melatonin in the treated-mice, with an increased activity of mitochondrial oxidative phosphorylation enzymes and a reduction of neuronal oxidative stress disorders.

Finally, two studies indicated that the administration of melatonin in mice was ineffective in protecting nigral dopaminergic neurons from MPTP-induced toxicity. Results for both studies indicated that the neuronal enzymatic levels and DA concentration were not different from melatonin-treated mice versus control [95,96].

### 4.12. Niacin (Vitamin B_3_)

B vitamins are a class of water-soluble vitamins that play important roles in cell metabolism, with a relevant role as enzyme cofactors in multiple biochemical pathways in all tissues. Good sources for B vitamins include legumes (pulses or beans), whole grains, potatoes, bananas, and above all meat. Probably the most popular use of vitamins is as metabolism booster, mainly for the athletes, and for the elderly which may need to supplement the intake of B vitamins because of problems in absorption and increased needs for energy production [97]. Among the vitamins B, niacin is an essential component for the human metabolism, its active form nicotinamide is the precursor of coenzymes NADH and NADPH, which are essential to produce ATP, and its deficiency leads to pellagra (Figure 4). Since a part of PD physiopathology is related to mitochondrial dysfunction and cellular energy failure, it was highlighted that niacin, due to its role in numerous metabolic pathways, has neuroprotective and antioxidant functions at low doses [83].

Jia et al. studied the effects of nicotinamide on mitochondrial function and oxidative stress in MPP^+^-induced cellular model of PD (1-methyl-4-phenylpyridinium, MPP^+^, a neurotoxin that acts by interfering with oxidative phosphorylation in mitochondria) [98]. Result showed that nicotinamide significantly protected human cells from an MPP^+^ toxicity, increasing cell survival and antioxidant generation, and reducing DNA damage.

Anderson et al. examined the potential of nicotinamide in a MPTP-induced mouse model of PD [99]. Among the different doses of nicotinamide administrated (125, 250, or 500 mg/kg i.p.), only the highest dose showed the recovery of striatal DA levels and neuroprotection in the animals treated with MPTP.

In clinical studies, a positive correlation was demonstrated between a high niacin diet and the reduced risk of PD [100]. Of interest is the case in which niacin, orally administrated for three months (500 mg twice daily), significantly improved rigidity and bradykinesia in a patient with idiopathic PD, though the original purpose was to treat hypertriglyceridemia [101]. Nevertheless, the adverse effects (unacceptable nightmares and skin rash), did not allow to prolong and better understand the framework of this result.

Finally, a population-based case-control study evaluated the nutrient intake as a risk factor for PD, failing in noticing a remarkable clinical efficacy of niacin though the study was conducted only in people aged ≥50 years in metropolitan Detroit [102]. More clinical observations are warranted to verify the efficacy as well as side effects of niacin in PD.

### 4.13. Vitamin C

Vitamin C (ascorbic acid, Figure 4) has an important role in the human body since it is involved in numerous physiological functions. Moreover, it is an excellent antioxidant, useful in reducing the presence of ROS, lipid peroxidation, and oxidative stress [103]. The main food sources of vitamin C are vegetables and fruits, especially citrus fruits and paprika; in recent years more and more food supplements rich in vitamin C have emerged in the market.

The antioxidant activity of vitamin C can have important implications in the management of PD, as confirmed by Sershen et al., which demonstrated in vivo that ascorbic acid (100 mg/kg) administration prior to MPTP treatment can reduce ROS derivatives involved in MPTP neurotoxicity [104].

Seitz et al. carried out a study on the possible correlation between vitamin C and L-DOPA/dopamine concentrations in an in vitro model of human nerve cells [105]. In particular, the addition of vitamin C to the culture medium showed an increased production of L-DOPA and dopamine, mainly due to both an enhancement at metabolic level and involving the gene expression of the enzymes responsible for producing L-DOPA and dopamine.

Finally, Hughes et al. examined the association between the intake of antioxidants such as vitamin C and the risk of developing PD [106]. Analyzing more than 1000 cases of patients with PD, it was observed that the intake of vitamin C can reduce the risk of PD. However, the authors were unable to confirm this correlation due to the absence of data relating to some years in the follow-up study.

### 4.14. 6-Shogaol (from Zingiber officinale)

*Zingiber officinale* is an herbaceous plant of the Zingiberaceae, native to the south-east Asia and it is widely used as a spice and in the folk medicine. Commercialized with the English name of ginger, it is cultivated throughout the tropical and subtropical area. It produces clusters of white and pink flower buds that bloom into yellow flowers, with juicy and fleshy rhizomes which contains the active ingredients of the plant: essential oil (mainly composed of zingiberene), gingerols, shogaol (main responsible for the pungent taste), resins, and mucilage.

Among these, 6-shogaol (Figure 4) highlighted interesting properties for the treatment of PD [107].

Park and collaborators studied the neuroprotective and anti-inflammatory properties of this compound in in vitro and in vivo models of PD [108]. Results showed that treatment with 6-shogaol of rat mesencephalic cells exposed to MPP^+^ prevented dopaminergic induced cell loss, showing a counted neuron cells of 98.37% ± 10.27% compared to the control group. In the same cell cultures, 6-shagaol was able to suppress MPP^+^-induced production of neuroinflammatory factors in a dose-dependent manner. In vivo studies demonstrated the capacity of 6-shagaol to reduce MPTP-induced movement impairment of mice compared to control, indicating that this ginger extract compound is able to protect dopaminergic neurons, to inhibit the PD inflammatory pathways and to ameliorate the typical PD motor symptoms.

Ha et al. evaluated the anti-inflammatory effects of 6-shogaol in primary microglia cells and in an in vivo systemic inflammatory model induced by LPS [109]. Results showed significant neuroprotective effects in vivo in transient global ischemia via the inhibition of microglia, indicating the positive attitude of ginger to the reduction on neuroinflammation, one of the main pathological mechanism of PD. 

### 4.15. β-Carotene

**Figure 5:**
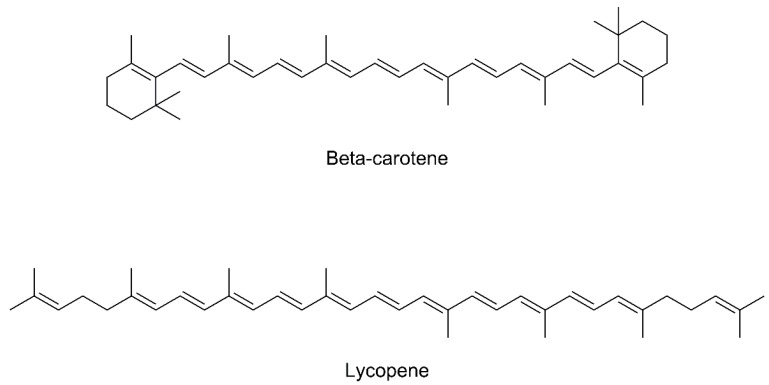
Chemical structures of β-carotene and lycopene.

β-carotene (provitamin A, Figure 5) is a vitamin A precursor, since it is composed of two retinyl groups, which can be broken down in the mucosa of the small intestine by β-carotene dioxygenase to form vitamin A. β-carotene can be found in yellow, orange, and green leafy vegetables and fruits (predominant in carrots, mango, maize, lentils, and spinach). As an antioxidant, it can scavenge singlet oxygen with very high efficiency, and it is also able to inhibit free radical reactions [110]. The very low toxicity of β-carotene and its potential efficacy in scavenging free radicals have led to several clinical trials assessing the antioxidant preventative activities, some of them also in the prevention and contrast to the PD disease.

Yang et al. prospectively related the consumption of dietary β-carotene with the antioxidant capacity and the reduced PD risk in two population-based cohorts (38.937 women and 45.837 men) [111]. Results suggested that the constant use of antioxidant supplements such as β-carotene can exert a protective effect on PD through the reduction of oxidative damage by neutralizing the effect of ROS.

Ono et al. assessed the in vitro potential anti-fibrillogenic and fibril-destabilizing activities of β-carotene, in a dose-dependent manner [112].

Finally, Etminan et al. studied the effect of β-carotene intake on the risk of PD through a meta-analysis of observational studies, but they could not highlight any protective effects from β-carotene, probably due to the small number of studies that included data on dietary intake of this carotenoid [113].

### 4.16. Lycopene

Lycopene is an acyclic isomer of beta-carotene, containing 11 conjugated and 2 non-conjugated double bonds, belonging to the carotenoid group (Figure 5). It is a natural compound that contributes to the red color of fruits and vegetables. It is found in tomatoes, watermelons, pink grapefruits, and apricots. Due to the high number of conjugated double bonds, lycopene is one of the most powerful natural antioxidants. Among the natural carotenoids, it demonstrated to possess in vitro the highest scavenger capacity against free radicals, 10-fold and 47-fold more effective in quenching singlet oxygen than alpha-tocopherol and β-carotene, and the ability to counteract ROS of 2-fold and 10-fold compared to the other antioxidants, respectively [114,115].

Kaur et al. assessed the potential antioxidant activity of lycopene on oxidative stress and neurobehavioral abnormalities in rotenone induced PD [116]. The authors administrated 10 mg/kg of lycopne orally to the rotenone-treated rats for 30 days, and results showed an increased activity by 39% compared to the control. Moreover, lycopene administration decreased the levels of malonilaldehyde, a measure of membrane lipid peroxidation, increased the levels of GSH by 75.35% and the level of superoxyde dismutase (SOD) by 12% when compared to control. This was accompanied by a partial inversion of cognitive and motor deficits in rotenone-treated rats.

Prema and collaborators investigated the neuroprotective activity of lycopene in an in vivo model of PD [117]. Administration of lycopene (5, 10, and 20 mg/kg per day, orally) protected MPTP-induced depletion of striatal DA in a dose-dependent manner. Several studies also indicated that the neuroprotective effect of lycopene could be related to the emendation of mitochondrial disfunction and neuroinflammation reduction, such as Sandhir et al., which investigated the neuroprotective effect of lycopene on an in vivo model of 3-nitropropionic acid-induced mitochondrial dysfunctions and oxidative stress [118]. Lycopene administration exhibited a protective effect on the induced mitochondrial dysfunctions and oxidative stress, with an important reduction in ROS formation and lipid peroxidation. These findings provide an evidence for the beneficial effects of lycopene supplementation, suggesting the therapeutic potential as neuroprotective and a strong antioxidant natural compound in the management of PD.

### 4.17. Flavonoids

**Figure 6:**
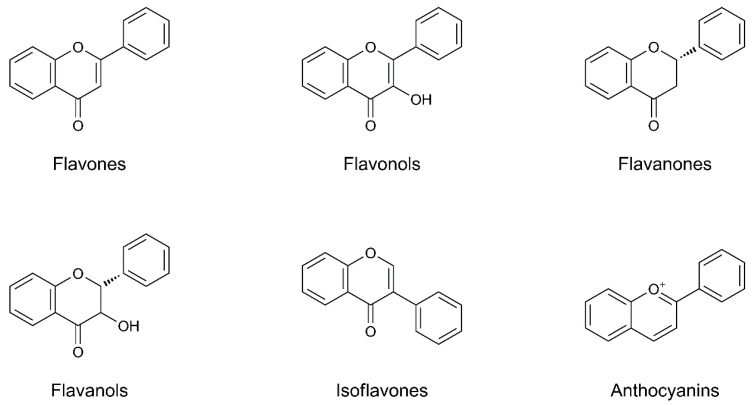
Chemical structures of flavonoids.

Flavonoids are a class of plant and fungus secondary metabolites, chemically based on a 15-carbon skeleton consisting of two benzene rings connected by a heterocyclic pyrone ring, divided into six subclasses based on their structural variations (Figure 6).

Common sources of flavonoids are several foods such as parsley, onions, blueberries and other berries, black tea, green tea, all citrus fruits, Ginkgo biloba, red wine, and dark chocolate. Alongside these common foods, a novel product recently re-evaluated for human nutrition as food supplement is pollen, which has proven to be a rich source of carbohydrates, lipids, proteins, vitamins, and minerals [119]. Moreover, pollen is one of the major sources of antioxidants, with a phenolic profile represented by more than 90% of the flavonoids (mostly glycosylated), which also represent the most abundant molecules among the nutrients that characterize its chemical composition [120].

Flavonoids can provide numerous health benefits due to their biologic effects, which include antioxidative, anti-inflammatory, antiapoptotic, and lipid-lowering properties, including a reduction in the risk of PD [121].

In 2018 Jung and Kim provided an overview of the scientific literature concerning the preventive and protective roles of flavonoids in the management of PD [122]. The authors, following the classification of flavonoids into the six major subclasses—flavones, flavonols, flavanones, flavanols, isoflavones, and anthocyanins—described different studies which support the relationship between the single molecules and PD. The main role that flavonoids can exert in the PD is the protection of neurons against oxidative stress, the capacity to suppress neuroinflammation, and the ability to interact with critical neuronal intracellular signaling pathways. These pathways involve protein kinase and lipid kinase signaling, which strongly affect neuronal function by altering the phosphorylation state of target molecules and by modulating gene expression. This suggests that flavonoids may be promising natural products for the prevention of PD and can potentially be employed as therapeutic compounds.

Among foods and food supplements that contain high quantities of flavonoids, there are some that are widely used in the daily diet, so that their constant intake can prevent the onset of PD or improve the clinical framework of patients who are already suffering from this disease.

#### 4.17.1. Quercetin (from Pollen)

Quercetin, (3,30,40,5,7-pentahydroxyl-flavone) is a natural flavonoid, present in most plants, fruits and vegetables such as broccoli, peppers, red onions, apples, and grapes. The major source is pollen, which presents quercetin in the glycosylated form as the predominant flavonoid among those present [123]. There are scientific evidences that quercetin possesses anti-inflammatory, antitumor, immunomodulating, and neuroprotective properties [124].

This last feature was confirmed in an in vivo study carried out by Kumar et al., which evaluated the neuroprotective effects of quercetin on mice treated with colchicine, which causes cognitive dysfunction and oxidative stress [125]. Chronic administration of quercetin showed clear improvements in the memory of mice, while biochemical assays on nerve cells showed a significant reduction in ROS and lipid peroxidation.

Sriraksa et al. confirmed the previous findings through an in vivo evaluation of the efficacy of quercetin in counteracting oxidative stress and memory deficits in 6-OHDA-lesioned rats [126]. Animals were treated with quercetin before and after the establishment of lesion, and they showed a marked improvement in cognitive functions, as well as a reduction of damage caused by induced oxidative stress compared to the control group.

#### 4.17.2. Epigallocatechin-3-Gallate (from Green Tea)

Green tea is a variant of tea obtained with non-treated leaves of *Camellia sinensis*. Like all commercialized teas, it has Chinese origins, and for centuries it has been consumed in various Asian regions, from Japan to the Middle East. In recent years, green tea has become more popular also in the rest of the world, mainly because the several health benefits it claims, including the prevention of risk cancer, cardiovascular diseases, diabetes, and neurodegenerative diseases [127,128]. Green tea is rich in flavonoids, among which one of the most important is (-)-epigallocatechin-3-gallate (EGCG), a compound part of the flavanols subgroup and that presents interesting antioxidant properties, which can contribute to counteracting the onset of PD [129].

Guo et al. evaluated from the histochemical point of view the neuroprotective effect of green tea on 6-OHDA-treated mouse model of PD, identifying a marked protection from ROS, a reduction in lipid peroxidation, and in the intracellular nitrite and nitrates levels [130].

Levites et al. demonstrated the potential free radical scavenger activity of polyphenols of green tea through an in vivo study on 6-OHDA mouse model of PD [131]. The results showed a reduction in oxidative stress and an inhibition of the activation of gene promoters that influence cell death in animals that received green tea extract prior to the treatment with 6-OHDA.

Hellenbrand et al. examined the possible correlation between the ingestion of green tea and the onset of PD, in a case-control study of the dietary habits of patients with PD compared to a control group [132]. The results showed a moderate lower risk of developing PD among people who regularly consume green tea in their diet.

Finally, Siddique et al. studied the role of EGCG on the transgenic *Drosophila* model of flies expressing normal human alpha synuclein (h-αS) in the neurons [133]. Results showed that supplementation with 0.25, 0.50, and 1.0 µg/mL of EGCG delayed locomotor dysfunction in a dose-dependent manner, as well as reduced the oxidative stress and apoptotic processes in the brain of the PD model flies.

#### 4.17.3. Ginkgo Biloba Extract

*Ginkgo biloba* is the only living species in the division Ginkgophyta, used for centuries in the traditional Chinese medicine. Its extracts, rich in flavonoids and terpenoids, may have beneficial effects in the management of PD [134].

Wu and Zu investigated the neuroprotective effect of *Ginkgo biloba* extract on MPTP-treated mice [135]. The pretreatment of animals with the extract, seven days before the administration of MPTP, significantly reduced the MPTP-induced neurotoxicity. A possible mechanism of neuroprotection was the antioxidant properties of flavonoids and ginkgolides against formation of free radicals. Moreover, fluorometric assays highlighted the capacity of the extract to inhibit in vitro the activity of monoamine oxidases B (MAO B), further reducing the degeneration or apoptosis of nigrostriatal dopaminergic neurons.

Rojas et al. confirmed the free radical scavenger activity of *Ginkgo biloba* extract in a mouse model of PD [136]. Mice treated prior or after 24 h the administration of MPTP showed a significant decrease in loss of striatal dopamine levels compared to the control. The authors also determined the oxidative stress levels by measuring the lipid peroxidation and the antioxidant enzymes activity. After the administration of *Ginkgo biloba* extract, the lipid peroxidation was reduced, whereas the activity of SOD, glutathione peroxidase (GPx), and glutathione reductase was enhanced, indicating that the neuroprotective action of the extract is related to the free radicals scavenging properties, the reduction of lipid peroxidation, and the capacity to stimulate several antioxidant enzymes.

## 5. Conclusions

Healthy ageing, primarily when a neurodegenerative disease is present, is possible by applying the correct pharmacological therapy, but diet and food supplementation often are a critical factor. The use of food supplements or functional food has undergone an enormous increment in recent years, with a wider market that offers diversified products for every type of need. The treatment of PD passes through a balanced diet, rich in biomolecules potentially useful to reduce the symptoms of the disease, by contrasting the mechanisms responsible for neurodegeneration. Moreover, a balanced diet and the use of food supplements based on vitamins, antioxidants, or elements with anti-inflammatory and neuroprotective properties can effectively act as a complement to the normal pharmacological therapies.

The molecules analyzed in this review have shown to actively contribute in countering the pathophysiological mechanisms of PD. Most of the molecules examined have a marked antioxidant capacity, important for combating the oxidative stress characteristic of PD. Other molecules are useful because they possess anti-inflammatory properties, or because they are able, through different molecular mechanisms, to induce the neuroprotection of dopaminergic neurons.

Nevertheless, time has a crucial role in the use of natural and/or plant-derived molecules, since usually they do not show any immediate action or tangible benefit in the short period, but constant use over a long period of time can bring several benefits in the treatment of several pathologies, in particular for slow-running neurodegenerative diseases such as PD.

## Figures and Tables

**Table 1:** Summary of the beneficial effects and the involved mechanisms for the examined compounds.

Molecule	Beneficial Effects	Mechanism	Ref.
oenzyme Q10	Antioxidant Neuroprotection	Coenzyme Q10, due to its 1,4-benzoquinone structure, is a powerful antioxidant acting as a free radical scavenger. Since it is also a redox component of the electron transport chain of mitochondria, it may exert neuroprotection through the modulation of mitochondrial activity in neuronal cells.	[44,45,46,47,48,49,50]
Lipoic acid	Antioxidant Anti-inflammatory Neuroprotection	The dithiolane ring, with its oxidized and reduced forms, makes lipoic acid a potent antioxidant. As an anti-inflammatory agent, it inhibits NF-kappaB and inflammatory cytokines like TNF-α. Neuroprotection is given by enhancing the intracellular levels of cysteine, thus increasing the glutathione levels.	[51,52,53,54]
*N*-acetyl-cysteine	Antioxidant Neuroprotection	The thiol group of *N*-acetyl-cysteine can act both as a direct antioxidant and as a glutathione precursor. It increases the mitochondrial complex I and IV activities and prevents reactive species of oxygen (ROS) accumulation in neuronal cells.	[55,56,57,58,59,60]
Vitamin E	Antioxidant	Vitamin E acts as a scavenger of several ROS by donating a hydrogen atom to free radicals, thus reducing their reactivity and toxicity.	[61,62,63,64,65,66]
Carvacrol	Antioxidant Anti-inflammatory Neuromodulation	Carvacrol induces the production of antioxidative enzymes and modulates oxidative stress. The anti-inflammatory effect is exerted by reducing the production of pro-inflammatory cytokines. Carvacrol is also able to inhibit the acetylcholinesterase activity, with positive effects on memory and cognitive performance in PD.	[67,68,69]
Curcumin	Antioxidant Anti-inflammatory Neuroprotection	Curcumin is an excellent free radical scavenger thanks to the phenolic rings and diketone groups. It protects mitochondrial complex I from enzyme nitration and subsequent inhibition, reducing mitochondrial disfunction. Anti-inflammatory and neuroprotective actions are exerted by modulation of chemokines which mediate the inflammatory cascade.	[70,71,72,73,74,75,76,77,78]
Omega−3 fatty acids	Antioxidant Anti-inflammatory	Omega-3 fatty acids reduce ROS formation acting as free radical scavengers. They also decrease chemotaxis of neutrophils and monocytes, as well as the production of pro-inflammatory cytokines.	[79,80]
Whey protein	Antioxidant	Since whey protein is an excellent source of cysteine, it can increase the production of glutathione, thus reducing oxidative stress.	[81,82]
Vitamin D_3_	Antioxidant Neuroprotection	Vitamin D_3_ inhibits oxidative stress, reduces free radical formation, and decreases neurotoxicity by enhancing autophagy signaling pathways. Neuroprotection is exerted by reducing the endothelial dysfunction observed in patients with PD.	[83,84,85,86,87]
Creatine	Antioxidant Neuroprotection	Creatine is able to contrast free radicals and ROS acting as antioxidant. Moreover, it can stimulate mitochondrial activity through the production of phosphocreatine, thus modulating the production of ATP and the energy homeostasis in the brain.	[88,89,90,91]
Melatonin	Antioxidant	Melatonin has interesting antioxidant properties, probably related to the indole group. The antioxidant activity is also performed by preventing the antioxidative catalysts lowering in neuronal cells.	[92,93,94,95,96]
Niacin (Vitamin B_3_)	Antioxidant Neuroprotection	Niacin and its active form nicotinamide reduce oxidative stress. Neuroprotection is reached since they are involved in the biosynthesis of nicotinamide adenine dinucleotide (NAD), an essential cofactor for the ATP production at the mitochondrial complex I level.	[97,98,99,100,101,102]
Vitamin C	Antioxidant	Vitamin C is an excellent antioxidant, suitable in reducing ROS levels, lipid peroxidation, and oxidative stress. It is also useful in regenerating other antioxidants.	[103,104,105,106]
6-shogaol	Antioxidant Anti-inflammatory Neuroprotection	The α,β-unsaturated ketone moiety makes 6-shogsol a good free radical scavenger. It possesses anti-inflammatory properties by reducing the production of prostaglandin E and pro-inflammatory cytokines such as TNF-α and interleukin-1β. Neuroprotection is assessed by inhibiting microglial activation.	[107,108,109]
β-carotene	Antioxidant	β-carotene is an excellent free radical scavenger. The high number of conjugated double bonds in its structure confers to this compound’s peculiar antioxidant properties.	[110,111,112,113]
Lycopene	Antioxidant	Lycopene is an excellent free radical scavenger. The high number of conjugated double bonds in its structure confers to this compound’s peculiar antioxidant properties.	[114,115,116,117,118]
Flavonoids *Quercetin* *Epigallocatechin-3-gallate* *Ginkgo Biloba extract*	Antioxidant Anti-inflammatory Neuroprotection Neuromodulation	The antioxidant activity of flavonoids depends upon the arrangement of functional groups on the 15-carbon skeleton. Beside the free radical scavenger capacity, they regulate the overproduction of inflammatory cytokines, reducing pro-inflammatory mediators and conferring to neuroprotection. This last property is exerted also through the increment of striatal dopamine and the modulation of cell survival/cell cycle genes, which increase neuronal survivability.	[119,120,121,122,125,126,127,128,134,135,136]
